# The Association Between Temperature Variability and Acute Coronary Syndrome Admissions: A Retrospective Study

**DOI:** 10.7759/cureus.108769

**Published:** 2026-05-13

**Authors:** Sabri Onur Caglar, Serdar Hira

**Affiliations:** 1 Cardiology, Private Bandirma Royal Hospital, Balıkesir, TUR; 2 Clinical Biochemistry, Private Bandirma Royal Hospital, Balıkesir, TUR

**Keywords:** acute coronary syndrome, cardiovascular diseases, climate, environmental exposure, hospital admissions, meteorological factors, retrospective study, risk factors, seasonal variation, temperature variability

## Abstract

Introduction

Meteorological factors have been suggested to influence acute coronary events, but the role of temperature variability remains incompletely understood. This study aimed to evaluate the association between meteorological parameters, particularly temperature variability, and hospital admissions for acute coronary syndrome (ACS).

Methods

This retrospective observational ecological time-series study included 1,376 patients who were hospitalized in two cardiology centers between January 2009 and December 2010. ACS was defined as the combination of ST-elevation myocardial infarction (STEMI), non-ST-elevation myocardial infarction (NSTEMI), and unstable angina pectoris (USAP). Meteorological data, including temperature and humidity parameters, were obtained from official records. Seasonal differences were analyzed, and the relationship between meteorological variables and ACS admissions was evaluated using correlation and linear regression analyses.

Results

A total of 981 patients were classified as having ACS. The highest number of ACS admissions was observed in May (n = 122), while the lowest occurred in September (n = 35). Total ACS admissions were similar during the cold season compared to the warm season (83.2 ± 21.3 vs. 80.3 ± 33.2, p = 0.86). In regression analysis, maximum humidity change (β = 0.122, p = 0.048), mean temperature (β = 0.813, p = 0.027), mean temperature change (β = -0.171, p = 0.025), minimum temperature change (β = 0.154, p = 0.016), and minimum humidity (β = -0.465, p = 0.014) were significantly associated with ACS admissions. Additionally, maximum temperature change (β = 0.886, p = 0.015) and daily temperature variation (β = -1.151, p = 0.004) showed significant associations with daily ACS admissions.

Conclusions

Although ACS admissions were numerically higher during colder periods, this difference did not reach statistical significance. Several temperature variability parameters were significantly associated with daily ACS admissions. These findings suggest that short-term fluctuations in temperature may be associated with ACS admissions, but causal relationships cannot be established because of the retrospective observational study design.

## Introduction

Cardiovascular diseases remain among the leading causes of morbidity and mortality worldwide, with acute coronary syndrome (ACS) representing a major contributor to hospital admissions and healthcare burden [1]. Numerous environmental and physiological factors have been implicated in the occurrence of acute coronary events, and meteorological conditions have received increasing attention [2]. Previous studies have demonstrated that seasonal variations, particularly colder temperatures, are associated with increased rates of myocardial infarction and cardiovascular mortality [3]. Temperature, humidity, and atmospheric pressure have been investigated as potential environmental contributors to cardiovascular events, although results remain inconsistent across populations and geographic regions [4].

Emerging evidence suggests that short-term fluctuations in temperature may also be associated with acute cardiovascular events [5,6]. Rapid changes in temperature may induce physiological stress and alter vascular tone, blood pressure, and sympathetic activity, potentially triggering acute coronary events [7,8]. In addition to environmental exposures, psychosocial and social determinants of cardiovascular health, including stress, depression, anxiety, socioeconomic conditions, and social isolation, are increasingly recognized as important contributors to cardiovascular risk [9,10].

Although several studies have evaluated seasonal and absolute temperature effects on cardiovascular outcomes, relatively few have specifically focused on short-term temperature variability using daily meteorological observations in regions with moderate climates. Accordingly, this study aimed to evaluate the association between meteorological parameters, particularly temperature variability, and hospital admissions for ACS.

## Materials and methods

Study design and population

This study was designed as a retrospective observational ecological time-series study. The study evaluated the association between daily ACS admissions and daily meteorological variables between January 1, 2009, and December 31, 2010, in the Cardiology Department of Düzce University Faculty of Medicine and Düzce Atatürk State Hospital.

A total of 1,376 patients were included in the study. Among the 1,376 hospitalized cardiovascular patients screened during the study period, 981 patients met the predefined ACS criteria (ST-elevation myocardial infarction (STEMI), non-ST-elevation myocardial infarction (NSTEMI), or unstable angina pectoris (USAP)) and were included in the final ACS analysis. The remaining 395 patients were hospitalized due to non-ACS cardiovascular conditions, including congestive heart failure (CHF), atrial fibrillation (AF), cardiac tamponade, and other cardiovascular diagnoses, and were therefore excluded from the ACS-specific analyses. Patients were categorized according to their clinical diagnoses as STEMI, NSTEMI, USAP, CHF, atrial fibrillation (AF), and cardiac tamponade. For the present study, ACS was defined as the combination of STEMI, NSTEMI, and USAP cases.

Inclusion and exclusion criteria

All adult patients (≥18 years) hospitalized with a diagnosis of cardiovascular disease between January 1, 2009, and December 31, 2010, were screened. Patients diagnosed with ACS, including STEMI, NSTEMI, and USAP, were included in the analysis. Patients with incomplete or missing clinical data, including missing admission dates or diagnostic classification, were excluded. Patients without corresponding meteorological data for the date of admission were also excluded. Additionally, patients admitted for non-cardiac conditions or those without a confirmed diagnosis of ACS were excluded from the analysis. Duplicate records and repeat hospitalizations occurring within the same hospitalization episode were also excluded to avoid data duplication. Furthermore, patients with uncertain diagnoses or conflicting medical records were excluded to ensure data accuracy and consistency.

Diagnostic criteria

The diagnosis of ACS and its subtypes was established according to the contemporary clinical guidelines valid during the study period (2009-2010), based on clinical presentation, electrocardiographic findings, and cardiac biomarker levels [11]. STEMI was defined as the presence of chest pain consistent with myocardial ischemia lasting ≥20 minutes, accompanied by persistent ST-segment elevation in at least two contiguous leads on electrocardiography and elevated cardiac biomarkers. NSTEMI was defined as the presence of ischemic symptoms with elevated cardiac biomarkers (troponin) in the absence of persistent ST-segment elevation. USAP was defined as ischemic chest pain at rest or with minimal exertion without elevation of cardiac biomarkers but with or without electrocardiographic changes suggestive of ischemia.

Meteorological data

Meteorological data for Düzce province during the study period were obtained from the General Directorate of Meteorology of Turkey (https://www.mgm.gov.tr) [12]. The parameters analyzed included mean temperature, temperature variability measures (mean, minimum, and maximum temperature changes), and humidity parameters. Seasonal comparisons were performed using monthly aggregated data, whereas correlation and regression analyses were conducted using daily data. In addition to absolute temperature values, temperature variability parameters were calculated, including the magnitude of temperature change between consecutive days.

Temperature variability was defined as the absolute difference between mean temperatures of consecutive days (ΔT = |Tₙ − Tₙ₋₁|). These variables were used to evaluate the potential effect of short-term temperature fluctuations on ACS admissions. Mean temperature change was defined as the absolute difference between the mean temperatures of two consecutive days. Minimum temperature change was defined as the absolute difference between the minimum daily temperatures of consecutive days. Maximum temperature change was defined as the absolute difference between the maximum daily temperatures of consecutive days. Daily temperature variation was defined as the difference between maximum and minimum temperatures recorded within the same day. Apparent temperature represented the perceived outdoor temperature obtained from the official meteorological records. Maximum humidity change was defined as the absolute difference between maximum daily humidity values of consecutive days. Meteorological observations were matched with corresponding daily ACS admission counts according to the calendar date.

Data processing

Monthly distributions of ACS admissions were calculated based on hospital records. For seasonal comparisons, months were grouped into two categories: cold season (October to March) and warm season (April to September). The number of hospital admissions for ACS and its subtypes was calculated for each month and season. Seasonal comparisons were performed using mean admission values and standard deviations (SD). Monthly aggregated data were used for descriptive seasonal comparisons, whereas daily admission counts and daily meteorological observations were used for correlation and regression analyses.

Ethics statement

This study was approved by the Ethics Committee of Düzce University (approval No: 2010/35, date: 29/07/2010). The study was conducted in accordance with the principles of the Declaration of Helsinki. The dataset used in this study was originally collected as part of the first author's academic thesis. Data collection was performed retrospectively using previously recorded hospital and meteorological records covering the period between January 2009 and December 2010. Ethical approval was obtained during the latter phase of the study period before data extraction and statistical analysis.

Statistical analysis

All statistical analyses were performed using SPSS Statistics software, version 13.0 (IBM Corp., Armonk, NY). Normality of continuous variables was assessed using the Kolmogorov-Smirnov test. Continuous variables were expressed as mean ± SD. Differences between seasonal groups were evaluated using appropriate comparative statistical tests according to data distribution. The relationship between meteorological variables and hospital admissions was assessed using Pearson correlation analysis. Multicollinearity was assessed using the variance inflation factor (VIF) values. To evaluate variables associated with ACS admissions, multivariable linear regression analysis was performed, including meteorological variables with a p-value <0.10 in univariate analysis. The dependent variable in the regression model was the daily number of ACS admissions.

Given the retrospective exploratory nature of the study and the aggregated structure of the available daily admission data, linear regression analysis was used as an exploratory approach to evaluate associative relationships between meteorological variables and ACS admissions. Because the analyses were based on retrospectively collected daily aggregated admission data, adjustments for seasonality, long-term temporal trends, autocorrelation, and lag effects were not performed. Therefore, the regression findings should be interpreted as associative rather than causal. A p-value of less than 0.05 was considered statistically significant.

## Results

Among the 1,376 hospitalized cardiovascular patients screened during the study period, 981 patients met the predefined ACS criteria and were included in the final analysis. Of these patients, 324 were diagnosed with STEMI, 373 with NSTEMI, and 284 with USAP. The monthly distribution of ACS admissions is presented in Table 1. The highest number of total ACS admissions occurred in May (n = 122), while the lowest was recorded in September (n = 35). STEMI admissions were most frequent in February (n = 43), NSTEMI admissions in May (n = 50), and USAP admissions in January (n = 35).

**Table 1:** Monthly distribution of ACS admissions ACS: acute coronary syndrome; STEMI: ST-elevation myocardial infarction; NSTEMI: non-ST-elevation myocardial infarction; USAP: unstable angina pectoris

Month	STEMI	NSTEMI	USAP	Total ACS
January	25	40	35	100
February	43	32	27	102
March	36	39	28	103
April	31	44	21	96
May	43	50	29	122
June	40	35	27	102
July	27	21	30	78
August	21	17	11	49
September	9	12	14	35
October	9	27	21	57
November	28	30	18	76
December	12	26	23	61
Total	324	373	284	981

Seasonal comparisons of ACS admissions are shown in Table 2. Mean STEMI admissions were 25.5 ± 13.8 in the cold season and 28.5 ± 11.8 in the warm season. Mean NSTEMI admissions were 32.3 ± 6.2 in the cold season and 29.8 ± 12.5 in the warm season. Mean USAP admissions were 25.3 ± 5.6 in the cold season and 22.0 ± 7.5 in the warm season. Total ACS admissions were 83.2 ± 21.3 in the cold season and 80.3 ± 33.2 in the warm season. Overall, only a modest numerical variation between seasons was observed.

**Table 2:** Descriptive comparison of mean daily hospital admissions during cold and warm seasons Appropriate comparative statistical tests were used according to data distribution. Seasonal comparisons are presented primarily for descriptive evaluation of admission patterns SD: standard deviation; STEMI: ST-elevation myocardial infarction; NSTEMI: non-ST-elevation myocardial infarction; USAP: unstable angina pectoris; ACS: acute coronary syndrome

Group	Cold season (Oct-Mar), mean ± SD	Warm season (Apr-Sep), mean ± SD	t-value	p-value
STEMI	25.5 ± 13.8	28.5 ± 11.8	-0.43	0.278
NSTEMI	32.3 ± 6.2	29.8 ± 12.5	0.44	0.511
USAP	25.3 ± 5.6	22.0 ± 7.5	0.97	0.144
Total ACS	83.2 ± 21.3	80.3 ± 33.2	0.2	0.86

The results of the multivariable linear regression analysis are presented in Table 3. Significant associations with daily ACS admissions were observed for maximum humidity change (β = 0.122, p = 0.048), mean temperature (β = 0.813, p = 0.027), mean temperature change (β = -0.171, p = 0.025), minimum temperature change (β = 0.154, p = 0.016), and minimum humidity (β = -0.465, p = 0.014). Additionally, maximum temperature change (β = 0.886, p = 0.015) and daily temperature variation (β = -1.151, p = 0.004) were also significantly associated with daily ACS admissions.

**Table 3:** Multivariable analysis of factors associated with daily ACS admissions Multivariable linear regression analysis was performed to evaluate associations between meteorological variables and daily ACS admissions. Variables with p<0.10 in univariate analysis were included in the model. Temperature variables are expressed in °C and humidity variables in %. Standardized β coefficients are presented to emphasize the direction and relative magnitude of associations. Regression findings should be interpreted as exploratory associations ACS: acute coronary syndrome; β: standardized regression coefficient

Variable	β coefficient	p-value	Interpretation
Maximum humidity change	0.122	0.048	Positive association
Mean temperature	0.813	0.027	Positive association
Mean temperature change	-0.171	0.025	Negative association
Minimum temperature change	0.154	0.016	Positive association
Minimum humidity	-0.465	0.014	Negative association
Maximum temperature change	0.886	0.015	Positive association
Daily temperature variation	-1.151	0.004	Negative association

**Figure 1:**
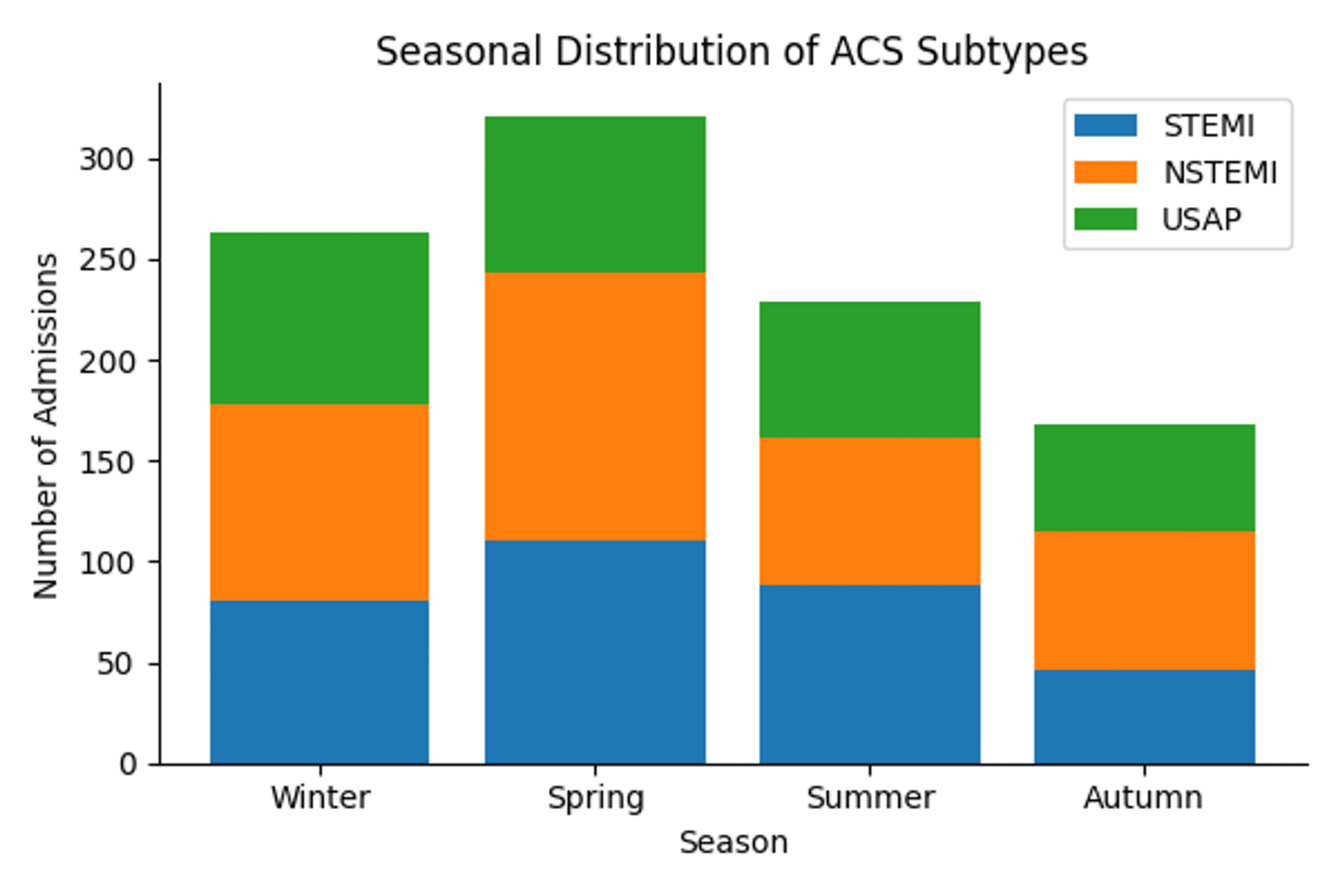
Seasonal distribution of acute coronary syndrome admissions (n = 981) Cold season: October-March; warm season: April-September. Seasonal definitions: winter (December-February), spring (March-May), summer (June-August), autumn (September-November) ACS: acute coronary syndrome; STEMI: ST-elevation myocardial infarction; NSTEMI: non-ST-elevation myocardial infarction; USAP: unstable angina pectoris

**Figure 2:**
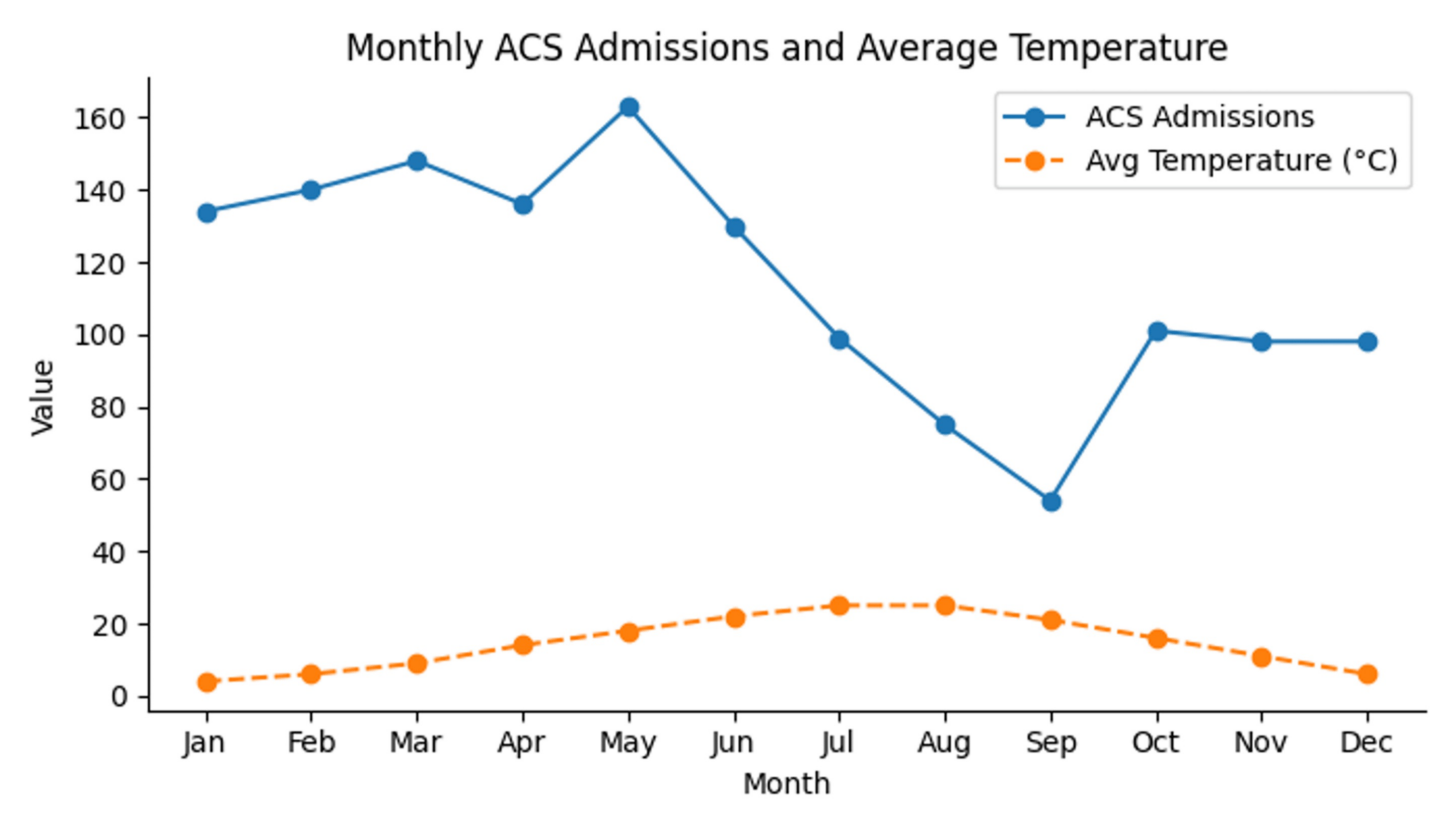
Monthly trend of ACS admissions and average temperature Temperature values represent monthly average ambient temperatures obtained from the Turkish General Directorate of Meteorology. Temperature variables are expressed in °C. The figure is presented for descriptive visualization purposes ACS: acute coronary syndrome

Pearson correlation analysis demonstrated weak but statistically significant associations between daily ACS admissions and several meteorological variables (Table 4). Maximum humidity change showed a weak positive correlation, whereas several temperature-related variables, including maximum temperature, apparent temperature, daily temperature variation, and minimum temperature, showed weak negative correlations. Multicollinearity analysis indicated that all variables had VIF values below 5, demonstrating no significant multicollinearity among the predictors included in the regression model (Table 5). The seasonal distribution of ACS admissions and its subtypes is shown in Figure 1, while the monthly trend of ACS admissions and average temperature is presented in Figure 2.

**Table 4:** Pearson correlation between daily meteorological variables and daily ACS admissions (n = 730 days) Pearson correlation analysis was performed using daily meteorological observations and corresponding daily ACS admission counts between January 1, 2009, and December 31, 2010 (n = 730 days). A p-value <0.05 was considered statistically significant. Temperature variables are expressed in °C, and humidity variables in % ACS: acute coronary syndrome; r: Pearson correlation coefficient

Variable	r	p-value
Maximum humidity	0.056	0.131
Maximum humidity change	0.085	0.022
Maximum temperature	-0.097	0.009
Apparent temperature (feels like)	-0.096	0.009
Daily temperature variation	-0.104	0.005
Minimum temperature	-0.094	0.011

**Table 5:** Multicollinearity assessment of variables included in the regression model Multicollinearity among independent variables was assessed using VIF. A VIF value greater than 5 was considered indicative of significant multicollinearity. Temperature variables are expressed in °C, and humidity variables in % VIF: variance inflation factor

Variable	VIF
Maximum humidity change	1.42
Mean temperature	2.31
Mean temperature change	1.88
Minimum temperature change	1.76
Minimum humidity	1.95
Maximum temperature change	2.64
Daily temperature variation	2.12

## Discussion

In this study, the relationship between meteorological parameters and ACS admissions was evaluated, with a particular focus on temperature variability. The main findings indicated that total ACS admissions were numerically higher during the cold season; however, this difference was not statistically significant. Unlike previous studies, which primarily focused on absolute temperature values, this study specifically evaluated the association between temperature variability and ACS admissions. Previous studies have reported an increased incidence of myocardial infarction and cardiovascular mortality during colder months [13]. Low ambient temperature has been associated with vasoconstriction, increased blood pressure, and enhanced sympathetic activity, all of which may contribute to acute coronary events [5,7]. In this study, some numerical seasonal variation was observed, but statistically robust and consistent differences across ACS subtypes were not demonstrated. This discrepancy may partly reflect the moderate climatic characteristics of the study region and the relatively limited subgroup sample sizes.

However, beyond seasonal differences, several temperature variability parameters demonstrated significant associations with ACS admissions in the regression analyses [14]. Both maximum temperature change and daily temperature variation were significantly correlated with daily ACS admissions. Interestingly, the peak in ACS admissions occurred in May rather than during the coldest months, suggesting that transitional periods between seasons may also be linked to increased cardiovascular admissions [15]. These findings are consistent with the hypothesis that short-term fluctuations in temperature contribute to physiological stress responses that may be associated with acute coronary events.

The potential mechanisms underlying this relationship may include abrupt changes in vascular tone, endothelial dysfunction, and alterations in blood viscosity in response to sudden temperature shifts [5,7]. The endothelium is a key regulator of vascular tone and hemodynamic balance through the release of vasoactive substances, such as nitric oxide and endothelin [16]. Disruption of this balance leads to endothelial dysfunction, characterized by oxidative stress, inflammation, and impaired vasodilation, all of which are strongly linked to the development of atherosclerosis and acute coronary events [8,17]. Such physiological responses may also contribute to plaque instability and thrombotic events in susceptible individuals.

The findings of this study are in line with previous reports suggesting that short-term environmental changes may influence cardiovascular events [2,5,18]. While many earlier studies primarily focused on absolute temperature, the present study suggests that dynamic temperature changes are also relevant in the evaluation of cardiovascular admissions [19]. The dataset used in this study has previously contributed to a broader analysis of meteorological variables and cardiac hospitalizations [20]. However, the present study represents a focused secondary analysis specifically assessing temperature variability parameters in relation to ACS admissions. Although some meteorological variables were included in the previous publication, the current study was designed with a distinct analytical focus on short-term temperature variability and its association with ACS admissions. By narrowing the scope of analysis, the present study provides a more targeted evaluation of the potential effects of dynamic temperature fluctuations.

Limitations and strengths

This study has several limitations. As a retrospective observational ecological time-series study, the findings are inherently susceptible to ecological fallacy, whereby associations identified at the population level may not accurately reflect relationships at the individual level. Furthermore, the retrospective observational design precludes causal inference and may be influenced by residual or unmeasured confounding factors. Accordingly, the findings should be interpreted with appropriate caution, and further prospective individual-level studies are warranted to validate these observations. Additionally, the study was conducted in a single geographic region, which may limit the generalizability of the findings.

The temperature values used in graphical representations were based on monthly averages and should be interpreted as descriptive rather than analytical variables. Potential confounders, such as air pollution, physical activity, and comorbid conditions, were not included in the analysis [21]. Moreover, psychosocial and socioeconomic factors- including stress, depression, anxiety, and other social determinants of health - were not evaluated in this study. Adjustments for seasonality, long-term temporal trends, autocorrelation, and lag effects were not performed. Therefore, interpretations based on these visual analyses should be made cautiously. Additionally, the use of aggregated admission data may have limited the ability to fully capture complex temporal relationships between meteorological variables and ACS admissions.

Despite these limitations, the findings of this study may offer clinically relevant insights into the potential impact of short-term temperature variability on ACS admissions [22]. From a clinical and public health perspective, monitoring periods of increased temperature variability could enhance awareness of environmentally associated cardiovascular risk, particularly in susceptible populations and in regions with moderate climatic variability. These findings may also inform future research exploring the incorporation of environmental parameters into cardiovascular risk assessment models.

## Conclusions

Although ACS admissions were numerically higher during colder periods, statistically significant and consistent seasonal differences were not observed across ACS subtypes. Several temperature variability parameters were significantly associated with daily ACS admissions in this retrospective ecological time-series study. These findings support a possible association between short-term temperature fluctuations and cardiovascular admissions; however, further prospective studies with more comprehensive adjustments for temporal and environmental confounders are needed to clarify these relationships.

## References

[1] Kraler S, Mueller C, Libby P, Bhatt DL (2025). Acute coronary syndromes: mechanisms, challenges, and new opportunities. Eur Heart J.

[2] Lv T, Liu Q, Wang Y, Zhang P (2025). Climate change and cardiovascular health: environmental stressors, mechanistic insights, and clinical perspectives. Rev Cardiovasc Med.

[3] Kuzmenko NV, Tsyrlin VA, Pliss MG, Galagudza MM (2022). Seasonal dynamics of myocardial infarctions in regions with different types of a climate: a meta-analysis. Egypt Heart J.

[4] Münzel T, Hahad O, Sørensen M, Lelieveld J, Duerr GD, Nieuwenhuijsen M, Daiber A (2022). Environmental risk factors and cardiovascular diseases: a comprehensive expert review. Cardiovasc Res.

[5] De Vita A, Belmusto A, Di Perna F, Tremamunno S, De Matteis G, Franceschi F, Covino M (2024). The impact of climate change and extreme weather conditions on cardiovascular health and acute cardiovascular diseases. J Clin Med.

[6] Rowland ST, Boehme AK, Rush J, Just AC, Kioumourtzoglou MA (2020). Can ultra short-term changes in ambient temperature trigger myocardial infarction?. Environ Int.

[7] Goel H, Shah K, Kumar A, Hippen JT, Nadar SK (2022). Temperature, cardiovascular mortality, and the role of hypertension and renin-angiotensin-aldosterone axis in seasonal adversity: a narrative review. J Hum Hypertens.

[8] Gostimirovic M, Novakovic R, Rajkovic J, Djokic V, Terzic D, Putnik S, Gojkovic-Bukarica L (2020). The influence of climate change on human cardiovascular function. Arch Environ Occup Health.

[9] Tosoratto J, López PJ, López-González ÁA, Sánchez CM, Rifá EM, Ramirez-Manent JI (2025). The influence of shift work on sociodemographic characteristics, anthropometric parameters, lifestyle behaviors, and its relationship with cardiovascular risk factors. Acad J Health Sci.

[10] Borkowski P, Borkowska N (2024). Understanding mental health challenges in cardiovascular care. Cureus.

[11] Thygesen K, Alpert JS, White HD (2007). Universal definition of myocardial infarction. Eur Heart J.

[12] (2026). Turkish State Meteorological Service (TSMS). https://www.mgm.gov.tr.

[13] Ni W, Stafoggia M, Zhang S (2024). Short-term effects of lower air temperature and cold spells on myocardial infarction hospitalizations in Sweden. J Am Coll Cardiol.

[14] Guo F, Do V, Cooper R (2021). Trends of temperature variability: which variability and what health implications?. Sci Total Environ.

[15] Licker M, Ellenberger C (2025). Impact of the circadian rhythm and seasonal changes on the outcome of cardiovascular interventions. J Clin Med.

[16] Wang X, He B (2024). Endothelial dysfunction: molecular mechanisms and clinical implications. MedComm (2020).

[17] Wang Z, Yang Y, Wang Q (2026). Pathological mechanisms and clinical research progress of endothelial dysfunction. Front Cardiovasc Med.

[18] Khraishah H, Alahmad B, Ostergard RL Jr (2022). Climate change and cardiovascular disease: implications for global health. Nat Rev Cardiol.

[19] Alahmad B, Khraishah H, Royé D (2023). Associations between extreme temperatures and cardiovascular cause-specific mortality: results from 27 countries. Circulation.

[20] Albayrak S, Çağlar O, Özhan H, Aslantaş Y, Ekinözü İ, Türker Y (2014). Meteorological variables and hospitalization due to cardiac causes. Konuralp Med J.

[21] Sagheer U, Al-Kindi S, Abohashem S (2024). Environmental pollution and cardiovascular disease: part 1 of 2: air pollution. JACC Adv.

[22] Olorunsogo TO, Ogugua JO, Muonde M, Maduka CP, Omotayo O (2024). Environmental factors in public health: a review of global challenges and solutions. World J Adv Res Rev.

